# INTESTINAL MALROTATION IN PATIENTS UNDERGOING BARIATRIC SURGERY

**DOI:** 10.1590/0102-6720201600S10007

**Published:** 2016

**Authors:** Eduardo Arevalo VIDAL, Francisco Abarca RENDON, Trino Andrade ZAMBRANO, Yudoco Andrade GARCÍA, Mario Ferrin VITERI, Josemberg Marins CAMPOS, Manoela Galvão RAMOS, Almino Cardoso RAMOS

**Affiliations:** 1 Department of General Surgery, Hospital Clínica Alcívar, Guayaquil, Ecuador;; 2 Federal University of Pernambuco, Recife, Brazil;; 3 Gastro-Obeso-Center Advanced Surgical Institute, São Paulo, Brazil.

**Keywords:** Obesity, Malrotation, Gastric bypass, Roux-en-Y, laparoscopic surgery

## Abstract

**Background:**

Intestinal malrotation is a rare congenital anomaly. In adults is very difficult to recognize due to the lack of symptoms. Diagnosis is usually incidental during surgical procedures or at autopsy.

**Aim:**

To review the occurrence and recognition of uneventful intestinal malrotation discovered during regular cases of bariatric surgeries.

**Methods:**

Were retrospectively reviewed the medical registry of 20,000 cases undergoing bariatric surgery, from January 2002 to January 2016, looking for the occurrence of intestinal malrotation and consequences in the intraoperative technique and immediate evolution of the patients.

**Results:**

Five cases (0,025%) of intestinal malrotation were found. All of them were males, aging 45, 49, 37,52 and 39 years; BMI 35, 42, 49, 47 and 52 kg/m^2^, all of them with a past medical history of morbid obesity. The patient with BMI 35 kg/m^2^ suffered from type 2 diabetes also. All procedures were completed by laparoscopic approach, with no conversions. In one patient was not possible to move the jejunum to the upper abdomen in order to establish the gastrojejunostomy and a sleeve gastrectomy was performed. In another patient was not possible to fully recognize the anatomy due to bowel adhesions and a single anastomosis gastric bypass was preferred. No leaks or bleeding were identified. There were no perioperative complications. All patients were discharged 72 h after the procedure and no immediate 30-day complications were reported.

**Conclusion:**

Patients with malrotation can successfully undergo laparoscopic bariatric surgery. May be necessary changes in the surgical original strategy regarding the malrotation. Surgeons must check full abdominal anatomical condition prior to start the division of the stomach.

## RESUMO

**Racional:**

Má-rotação intestinal é rara anomalia congênita em adultos de difícil reconhecimento devido à falta de sintomas. O diagnóstico é feito geralmente incidentalmente durante procedimentos cirúrgicos ou durante autópsia.

**Objetivo:**

Verificar a ocorrência e reconhecimento não eventual da má-rotação intestinal durante operações bariátricas sequenciais.

**Método:**

Retrospectivamente foram analisados os prontuários médicos de 20.000 casos de operações bariátricas de janeiro 2002 a janeiro de 2016, procurando por ocorrência de má-rotação intestinal, sua interferência na técnica operatória aplicada e a evolução imediata no pós-operatório.

**Resultados:**

Foram encontrados cinco casos (0.025%) de má-rotação intestinal em homens com idades de 37, 39, 45, 49 e 52 anos e IMC de 35, 42, 49, 47 e 52 kg/m^2^. O paciente com IMC de 35 kg/m^2^ também sofria de diabete melito tipo 2. Todos os procedimentos foram realizados através de laparoscopia, sem conversões. Em um paciente não foi possível mover o jejuno para o abdome superior a fim de realizar gastrojejunostomia; foi, então, realizada gastrectomia vertical. Em outro paciente, não foi possível reconhecer totalmente a anatomia devido às aderências intestinais e foi decidido realizar bypass gástrico com anastomose única. Nenhum vazamento ou sangramento foi identificado. Não houve nenhuma complicação pós-operatória. Todos os pacientes foram liberados 72 h após o procedimento e nenhuma complicação foi notificada nos primeiros 30 dias.

**Conclusões:**

A cirurgia bariátrica laparoscópica pode ser realizada com sucesso em pacientes com má-rotação. Mudanças talvez sejam necessárias com relação à anomalia. Os cirurgiões devem verificar toda a condição anatômica abdominal antes de iniciar a secção gástrica.

## INTRODUCTION

Intestinal malrotation is a rare congenital anomaly that originates from failure of the normal rotation and fixation of the midgut during the embryologic development^4^ ^,^ ^7^ ^,^ ^8^ ^,^ ^11^ ^,^ ^14^ ^,^ ^16^. It usually appears during the neonatal period or the first year of life. In adults is a very rare condition and it is more difficult to recognize due to the lack of symptoms^4^ ^,^ ^7^ ^,^ ^16^. Diagnosis of intestinal malrotation during adult life is usually incidental during surgical procedures or at autopsy^4^ ^,^ ^7^ ^,^ ^14^. 

The prevalence of morbid obesity continues to increase around the world. Medical therapies for weight reduction are unsuccessful at achieving and maintaining weight loss in the obese population mainly in cases of severe obesity. Bariatric surgery continues to be the only method to achieve weight loss for most patients. Laparoscopic Roux-en-Y gastric bypass (RYGB) is one of the most common procedures performed for severe obesity^1^ ^,^ ^3^ ^,^ ^10^ ^,^ ^21^. 

Anatomic variations are uncommon, but can be found incidentally during surgery; surgeons need to be ready and alert in order to identify these anomalies that can require an alternative operative approach and technical adjustments^1^. The RYGB involves moving the first part of the jejunum to the upper compartment of the abdomen in order to perform the gastrojejunostomy^3^ ^,^ ^10^. In case of intestinal malrotation this step of the procedure could be difficult or even leads to change the original intention of the surgeon changing the technique for an exclusive approach of the stomach with the laparoscopic sleeve gastrectomy. 

The aim of this study was to review the occurrence and recognition of uneventful intestinal malrotation discovered during regular cases of bariatric surgeries where the original intention of the surgeon would be to submit the patients to RYGB; the necessary changes in the original procedure; and immediate postoperative evolution of the patients. 

## METHOD

The medical records of 20,000 cases underwent to bariatric surgery allegedly with the aim of undergoing a RYGB, during the time interval from January 2002 to January 2016, were reviewed looking for the occurrence of intestinal malrotation and consequences in the intraoperative technique and immediate evolution of the patients. All cases were recognized as standard indication to bariatric surgery with BMI over 40 kg/m^2^ or BMI over 35 kg/m^2^ with type 2 diabetes and had regular preoperative bariatric preparation with multidisciplinary group. 

## RESULTS

Among the 20,000 cases were found five cases of intestinal malrotation (0,025%). All of them were males, aging 37, 39, 45, 49 and 52 with prior clinical management for morbid obesity. The patient with BMI 35 kg/m^2^ suffered also of diabetes. The procedures were completed in reverse Trendelenburg position by laparoscopic approach, with no conversions, using five trocars technique with the surgeon working in between legs.

The stomach was visualized in its normal anatomical position and a 30 ml capacity gastric pouch was created without difficulty. After creating the gastric pouch the abdomen was explored, showing the ileoceal valve, appendix, cecum and right colon on the left side of the abdomen and the intestinal malrotation was suspected. The pylorus, duodenum and proximal jejunum were identified easily without any evidence of Ladd's band or other adhesions in the right upper abdominal quadrant. Was then identified the duodenojejunal ligament and run the small bowel in order to confirm that laparoscopic RYGB could be carried out. Once the anatomy was clarified an uneventful construction of a laparoscopic RYGB was completed. In the patient with BMI 52 kg/m^2^ was not possible to move the jejunum to the upper abdomen in order to establish the gastrojejunostomy. The stomach was reconstructed with a gastrogastric linear stapler reconnection and a sleeve gastrectomy performed thereafter. In another patient was not possible to fully recognize the anatomy due to bowel adherences and a single anastomosis gastric bypass was preferred. No leaks or bleeding were identified. There were no perioperative complications. All patients were discharged 72 h after the procedure and no immediate 30-day complications were reported.

## DISCUSSION

**FIGURE 1:**
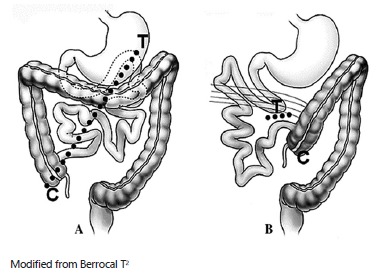
Intestinal malrotation: A) normal situation of the bowel; B) malrotation (proximal small bowel is on the right and cecum is on the left)

Intestinal malrotation is a rare congenital anomaly resulting from incomplete rotation and fixation of the midgut around the axis of the superior mesenteric artery. This typically results in nonrotation of the small bowel, with the small bowel located in the patient's right side of the abdomen and the colon on the left side of the abdomen (Figure 1)^4^ ^,^ ^7^ ^,^ ^8^ ^,^ ^11^ ^,^ ^14^ ^,^ ^16^. 

Three types of malrotation have been described^2^. Type I, occurs when normal midgut rotation ceases at six weeks, after 90^o^ of rotation; the proximal small bowel is on the right and cecum is on the left. Type II, the malrotation occurs between six and ten weeks and disrupts duodenal rotation. Type III, an error after ten weeks, in which the duodenum only completes 90^o^ of additional rotation. Fibrous bands called Ladd's bands crosses over the second portion of the duodenum connecting the cecum to the right upper quadrant^9^ ^,^ ^17^ ^,^ ^18^ ^,^ ^19^. 

Most patients present the anatomic defect within the neonatal period or the first year of life with symptoms of bowel obstruction, such as bilious vomiting, abdominal pain and distention, due to obstruction from Ladd's bands or midgut volvulus^4^ ^,^ ^6^ ^,^ ^7^ ^,^ ^15^ ^,^ ^16^ ^,^ ^20^. 

Diagnosis of intestinal malrotation during adult life is extremely uncommon. Studies that may be used to establish the diagnosis include CT scan, upper gastrointestinal series, and ultrasound^4^ ^,^ ^7^ ^,^ ^13^ ^,^ ^15^ ^,^ ^16^. Occasionally, malrotation can be asymptomatic or discovered incidentally during surgical procedures or at autopsy^4^ ^,^ ^7^ ^,^ ^14^ ^,^ ^20^.

The procedure of choice for symptomatic intestinal malrotation is Ladd procedure^4^ ^,^ ^5^ ^,^ ^7^ ^,^ ^13^ ^,^ ^15^ ^,^ ^16^. It mobilizes the right colon dividing the Ladd band, mobilizes the duodenum dividing the adhesions around the duodenum to broaden the mesentery base, and is performed prophylactic appendectomy because of the atypical localization. The laparoscopic approach to the Ladd's procedure is safe and effective, and promotes reduction of pain, ileus, length of hospital stay, and better cosmetic aspect^9^ ^,^ ^12^ ^,^ ^17^. 

In this series, bariatric surgery confirmed that the anomaly is rare occurring in 0,025% of the patients and in all cases the intestinal malrotation was type I with incidental diagnosis during the procedure; all surgeries were accomplished laparoscopically. Construction of the laparoscopic gastric bypass anatomy and technical orientation can vary and would require an alternate operative approach requiring technical adjustments^14^. For surgeons who usually begin surgery by making the gastric pouch and then pass to the intestinal part of the procedure, the recognition of an altered anatomy as an intestinal malrotation could be an unpleasant surprise leading to important changes in the original idea of the surgery or even to the abortion or conversion of the operation from one procedure to another. In this series was necessary to modify the technique in two cases. This must be a reason important enough for a proper review of the abdominal cavity and identification of anatomic variations prior to the start the stapling of the stomach. 

## CONCLUSION

Intestinal malrotation is a rare congenital condition where an abnormal rotated bowel is usually abnormally fixated and will be discovered incidentally during surgical procedures or at autopsy. Surgeons need to be ready in order to identify these anomalies that could require an alternative operative approach and technical adjustments. Patients with malrotation can successfully undergo laparoscopic bariatric surgery. In order to avoid unpleasant surprises and conditions that may hinder, increase the surgical time or even result in converting a procedure to another during surgery, surgeons must check full abdominal anatomical condition prior to start the operation.
